# On-treatment biomarkers can improve prediction of response to neoadjuvant chemotherapy in breast cancer

**DOI:** 10.1186/s13058-019-1159-3

**Published:** 2019-06-14

**Authors:** Richard J. Bownes, Arran K. Turnbull, Carlos Martinez-Perez, David A. Cameron, Andrew H. Sims, Olga Oikonomidou

**Affiliations:** 1 University of Edinburgh Cancer Research UK Centre, MRC Institute of Genetics and Molecular Medicine, Edinburgh, UK; 2 Edinburgh Cancer Centre, Western General Hospital, Edinburgh, UK

**Keywords:** Breast cancer, Chemotherapy, Gene expression, Response, Outcome, Predict, Neoadjuvant, Biomarker

## Abstract

**Background:**

Neoadjuvant chemotherapy is increasingly given preoperatively to shrink breast tumours prior to surgery. This approach also provides the opportunity to study the molecular changes associated with treatment and evaluate whether on-treatment sequential samples can improve response and outcome predictions over diagnostic or excision samples alone.

**Methods:**

This study included a total of 97 samples from a cohort of 50 women (aged 29–76, with 46% ER+ and 20% HER2+ tumours) with primary operable breast cancer who had been treated with neoadjuvant chemotherapy. Biopsies were taken at diagnosis, at 2 weeks on-treatment, mid-chemotherapy, and at resection. Fresh frozen samples were sequenced with Ion AmpliSeq Transcriptome yielding expression values for 12,635 genes. Differential expression analysis was performed across 16 patients with a complete pathological response (pCR) and 34 non-pCR patients, and over treatment time to identify significantly differentially expressed genes, pathways, and markers indicative of response status. Prediction accuracy was compared with estimations of established gene signatures, for this dataset and validated using data from the I-SPY 1 Trial.

**Results:**

Although changes upon treatment are largely similar between the two cohorts, very few genes were found to be consistently different between responders and non-responders, making the prediction of response difficult. AAGAB was identified as a novel potential on-treatment biomarker for pathological complete response, with an accuracy of 100% in the NEO training dataset and 78% accuracy in the I-SPY 1 testing dataset. AAGAB levels on-treatment were also significantly predictive of outcome (*p* = 0.048, *p* = 0.0036) in both cohorts. This single gene on-treatment biomarker had greater predictive accuracy than established prognostic tests, Mammaprint and PAM50 risk of recurrence score, although interestingly, both of these latter tests performed better in the on-treatment rather than the accepted pre-treatment setting.

**Conclusion:**

Changes in gene expression measured in sequential samples from breast cancer patients receiving neoadjuvant chemotherapy resulted in the identification of a potentially novel on-treatment biomarker and suggest that established prognostic tests may have greater prediction accuracy on than before treatment. These results support the potential use and further evaluation of on-treatment testing in breast cancer to improve the accuracy of tumour response prediction.

**Electronic supplementary material:**

The online version of this article (10.1186/s13058-019-1159-3) contains supplementary material, which is available to authorized users.

## Introduction

Chemotherapy is among the most common effective treatments for breast cancer, alongside radiotherapy, hormone therapy, and targeted treatments. Neoadjuvant chemotherapy is given prior to surgery with the aim to reduce the tumour burden and to provide early information on the response to treatment [1]. Studies have shown patients with tumours that have a pathological complete response (pCR) following neoadjuvant chemotherapy are much less likely to recur than those in women with residual disease [2]. Neoadjuvant chemotherapy is now considered as the standard of care in breast cancer and has seen a rise in recent years with data from powered studies suggesting that the pathological complete response achieved following neoadjuvant chemotherapy might be a surrogate of good prognosis [3]. A recent meta-analysis also showed significant tumour response and an increase in the rate of breast-conserving surgery following NACT with good rates of long-term local recurrence (5.5% vs. 15.9% adjuvant chemotherapy), however with an increase in the rate of short-term local relapses (1.35 RR 0–4 years, 1.53 RR 5–9 years) [4].

**Fig. 1:**
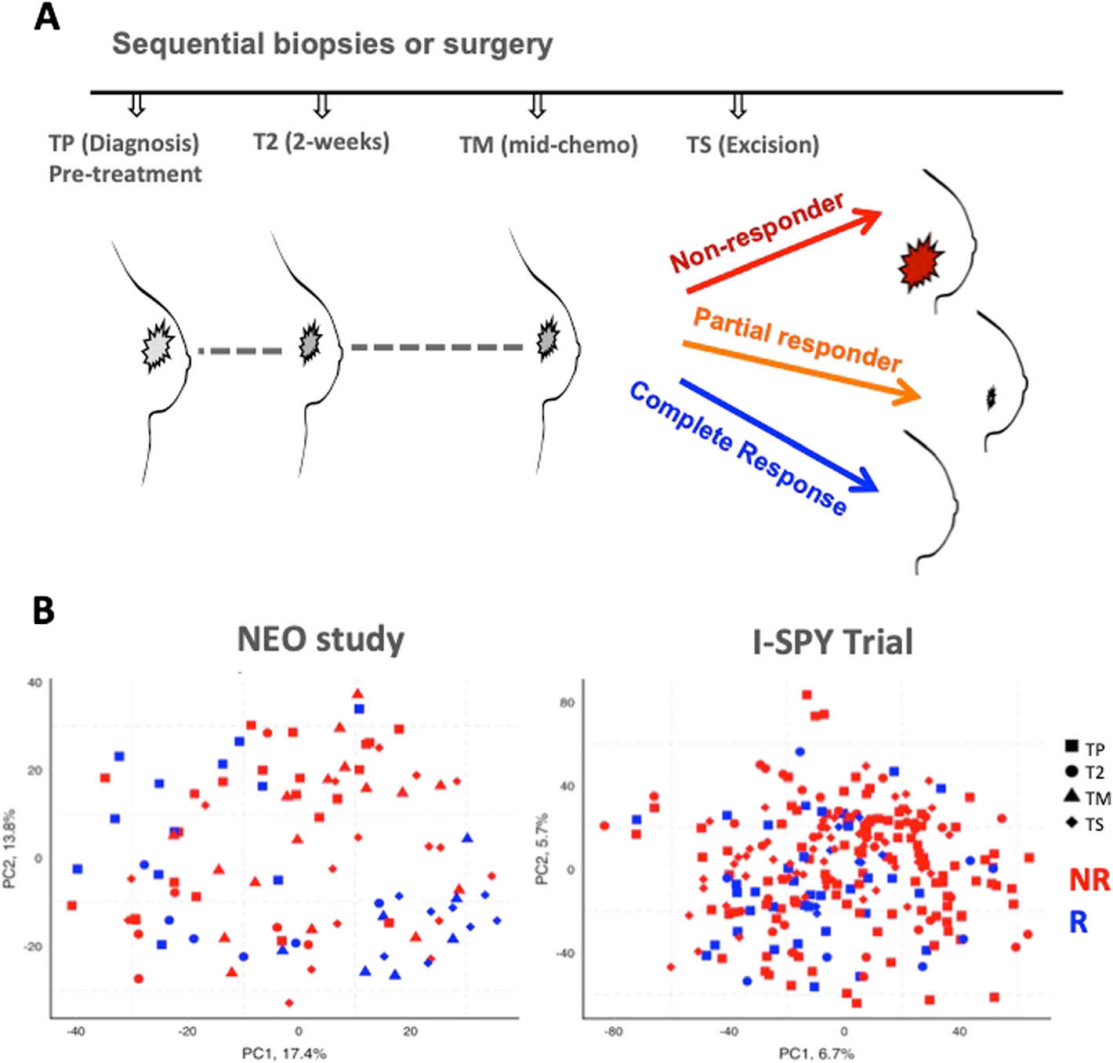
Unsupervised analysis cannot distinguish pre- and on-chemotherapy samples of breast tumours. **a** Schematic representation demonstrating sequential sampling of breast tumours during treatment. **b** PCA analysis of pre- and on-treatment samples from the Edinburgh NEO and I-SPY studies revealed no significant clustering of patients by time or response group. Red = non-responder, orange = partial responder, blue = complete responder

Neoadjuvant treatment provides a “window of opportunity” (Fig. 1a), where sequential sampling of a tumour enables observation of the changes that occur in response to treatment to be measured and considered in the context of response and outcome [5]. Neoadjuvant therapy studies and pre-surgical treatments allow for a unique in vivo analysis of tumour treatment response [6], as well as the possibility of predicting the response to treatment earlier in the treatment [5]. It has been suggested that on-treatment biomarkers may be superior to those measured before exposure to treatment [3, 7]. On-treatment information has already been shown to be informative for the accurate prediction of response to endocrine therapy [8]. Here, it was found that patients with elevated Ki67 levels (higher than 10%) at 2 or 4 weeks exhibited resistance to endocrine therapy and were triaged to neoadjuvant chemotherapy [8]. We have also demonstrated the potential of on-treatment biomarkers by developing a four-gene signature which combined pre-treatment expression levels or two biomarkers (IL6ST and NGFRAP1) with patient-matched 2-week on-treatment expression levels of two proliferation markers (ASPM, MCM4) to accurately predict the response to endocrine therapy in a blinded independent validation set [7].

Gene expression-based studies of neoadjuvant chemotherapy treatment to date have largely been limited to studying the association of pre-treatment samples with pathological response [9, 10]. Patient-matched sequential sampling gene expression studies have been previously attempted; however, they have not evaluated the predictive capacity or proposed new on-treatment predictive biomarkers [11–13].

In this study, we present the largest sequentially sampled patient-matched analysis of neoadjuvant chemotherapy-treated breast cancer tumours to evaluate whether on-treatment biomarkers can improve the accuracy of predicting response before resection. Numbers of patients with sequential breast tumour samples are limited, but we compare and validate our results with the data from the I-SPY 1 Trial.

## Materials and methods

### Patients, response criteria, and samples

The NEO study consists of 50 breast cancer patients with sequentially sampled biopsies at four time points, pre-treatment (PT, 34 samples), 2 weeks on treatment (T2, 12 samples), mid-chemo (TM, 23 samples), and at surgical resection (TS, 24 samples) with three clinically defined response statuses: complete responders (pCR by resection), good responders (tumour volume reduction, but lack of pCR), and non-responders (progressive disease or small tumour volume changes on treatment). Patients were of mixed histological grade and HER2 status; ages ranged from 29 to 76. Patients were primarily treated with 3 cycles of FEC and docetaxel with Herceptin where appropriate. Three patients received paclitaxel, one patient received additional carboplatin, one patient received Epi-cyclophosphamide and paclitaxel, and one patient received docetaxel and cyclophosphamide. Eligible patients were women with histologically confirmed invasive breast tumours and with no evidence of distant metastatic disease, no prior history of malignancy, and fit enough to receive chemotherapy in the opinion of the responsible clinician irrespective of age. All cases were discussed at the breast MDM in Edinburgh Breast Unit at the Western General Hospital, and consensus from this meeting was to be treated with neoadjuvant chemotherapy.

Core needle (16-gauge) biopsies were taken from the primary breast tumours before treatment (PT) and between 10 and 14 days after the first dose (T2) of chemotherapy. A third sample was taken at the mid-chemotherapy point day 20–21 (TM), and finally, a core biopsy was taken from the excision specimen (TS) after it has been removed prior to submission to pathology. Fixed and frozen samples of normal and tumour tissue were collected from all specimens.

### Gene expression profiling

RNA extraction was performed via Ribo0-RNAseq, and whole transcriptome sequencing was performed with Life Sciences Ion AmpliSeq™ Transcriptome Human Gene Expression Kit. This generated greater than 8 M reads per sample with an average of more than 90% valid reads for 12,365 targeted genes. Most analyses were performed in R (http://www.r-project.org) using packages available through CRAN (http://cran.r-project.org/) and Bioconductor (http://www.bioconductor.org/). Outside of the R environment, the stand-alone application Multiple Experiment Viewer (http://mev.tm4.org/) was utilised for pairwise ranked product feature selection, and DAVID (https://david.ncifcrf.gov/) was used for pathway identification. Additionally, the python package scikit-learn [14] was used for unsupervised clustering analysis. Ninety-seven samples were analysed over 13 AmpliSeq chips, but no systematic batch effects were evident and no batch correction was performed within the training data. Gene expression data for the NEO study has been made publicly available at the NCBI GEO data repository under accession GSE122630.

The I-SPY 1 Trial is composed of patients with invasive breast cancer > 3 cm, or at least one tumour-positive axillary lymph node [11]. Patients were treated with an anthracycline-based chemotherapy followed by taxanes [11]. Samples were normalised and corrected for background red/green signal; Bioconductor R packages marray and limma [15] were used to this end. From the original 221 patients, only 36 had matching pre- and on-treatment samples, and 39 had matching biopsy and excision samples; pathological complete response was used for response criteria. Pairwise gene expression was handled with SAM and follow-up analysis with Ingenuity Pathway Analysis from QIAGEN Bioinformatics. I-SPY 1 Trial data is hosted at NCBI GEO under accession GSE32603 [11].

### Statistical analysis methods

Principal component analysis (PCA) was performed on unsupervised gene lists to reduce dimensionality and visualise differences in response at all times and to identify present differences between patient treatment statuses. Local Fisher discriminant analysis (LFDA) [16] was used at each time point to determine if the response groups could be distinguished with treatment time with a semi-supervised clustering approach, concurrently with class advised *K*-means clustering. LFDA is a form of supervised dimensionality reduction that maximises between-class scattering and minimises within class scatter, and is a refined version of normal Fisher discriminant analysis [16]; this exploratory analysis was used in order to visualise comparative differences in treatment time, not as a means of feature selection. Pair-wise significance analysis of microarrays [17] using the *siggenes* package in R was used to consider the consistency of differentially expressed genes due to treatment in the sequential patient-matched samples. Rank Product analysis was used to identify differentially expressed genes between response classes at each time point. Successive levels of standard *p* value (0.05, 0.01, 0.001), without correction for multiple testing, were used in order to determine the number of differentially expressed genes, and at lower *p* values which the time points had the most strongly differentiating genes. Significance analysis of microarrays was also performed using varying false discovery rates (1%, 5%, 10%) to try to identify common differentially expressed genes between responders and non-responders across both datasets at each time point. Gene score enrichment analysis was used to validate the time point selection by looking for the highest number of enriched pathways. The gene list from the most differential time point (TM) using the NEO dataset was extracted and used in a random forest model (10,000 trees, m-try as the square root of the feature number) using pCR status as the class label (clinician-identified pCR and non-pCR). The most deterministic genes for class prediction were fed into a classification and regression tree in order to produce a maximally reduced and repeatable model; this methodology is further described by Turnbull et al. [7]. The CART decision tree was applied to the NEO dataset for training and tested in the independent I-SPY 1 dataset using the same cut-points determined by mean-centring the datasets. This protocol was repeated using the gene list from the pre-treatment only samples, using the same *p* values and tree configurations for selection. Survival analysis was performed at different time points using the log-rank test. Intrinsic subtypes, Mammaprint, and risk or relapse scores were estimated from the gene expression data using the *GeneFu* R package [18].

## Results

### Gene expression differences between responding and non-responding breast cancer tumours treated with chemotherapy are subtle and time dependent

**Fig. 2:**
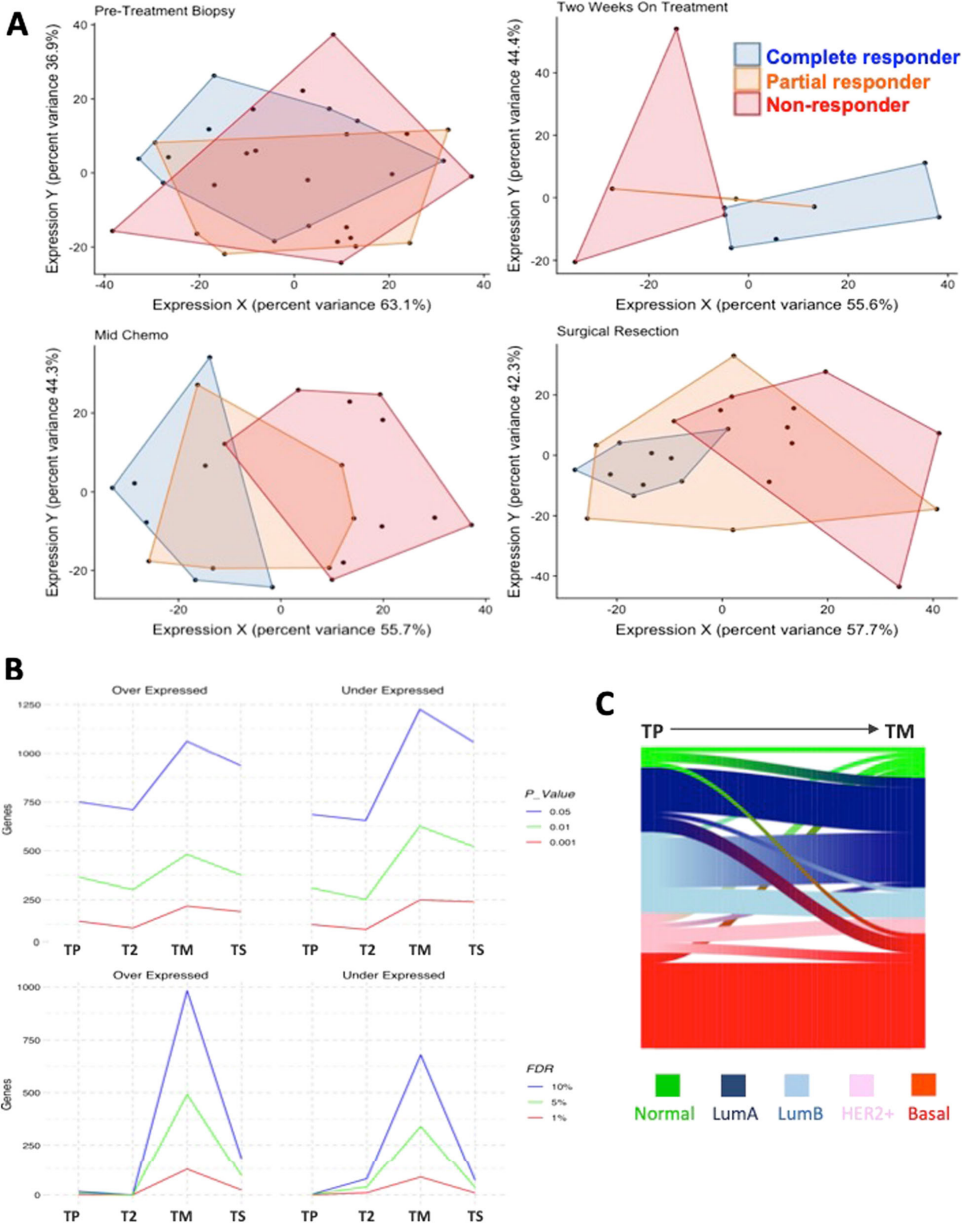
Responders and non-responders are more distinct on than before treatment. **a** Supervised clustering using local Fisher discriminant analysis (LFDA) indicates that as early as 2 weeks on treatment, there is a visible separation of the response classes that were unseen in the pre-treatment samples in the NEO dataset. Red = non-responder, orange = partial responder, blue = complete responder. **b** Greater numbers of genes are under and overexpressed between responders and non-responders on treatment. The three lines represent different statistical thresholds (*p < 0.05, **p < 0.01, and ***p < 0.001 or FDR = 10%, FDR = 5%, and FDR = 1%, gene lists are in Additional file 4: Tables S2 and S3) in the NEO dataset. **c** Sankey diagram illustrating the proportions of tumours that change or maintain PAM50 intrinsic subtype during chemotherapy treatment. Whilst basal subtypes remain mostly stable, the composition of the cohort changes with treatment time, which may help to identify responsive or non-responsive patients. PT = pre-treatment, T-ON = on-treatment

Unsupervised principal component analysis was first used to assess whether sequential patient-matched samples from patients receiving chemotherapy (Fig. 1b) would cluster by time point or response status. There was no significant grouping of patients according to sampling time: pre, early, or later after chemotherapy in either the NEO or I-SPY 1 studies (Fig. 1b). There were no significant differences between the two cohorts in terms of age, grade hormone receptor, and HER2 status, and the subset of patients with mid-chemo samples was not significantly different from the whole NEO cohort (Table 1). Patient-matched samples enable the pairwise analysis to look for consistent changes in the gene expression during treatment. Pairwise significance analysis of microarray analysis using a 10% false discovery rate (FDR) identified a relatively small proportion of overlapping upregulated (5%) and downregulated (4%) genes between the two studies. However, genes that were increased or decreased in response to treatment in one study were also clearly and consistently increased or decreased in the other study (Additional file 1: Figure S1A), further suggesting it would be difficult to discriminate responders from non-responders. Indeed, there was no clustering by response status before or during treatment (Additional file 1: Figure S1B). These results likely reflect the considerable inter-patient differences being substantially larger and more significant than the subtler commonalities in gene expression of a particular time point or response class of each tumour. More encouragingly, semi-supervised LFDA of each time point revealed significant separation on-treatment that was not apparent in pre-treatment samples; this indicated that there are meaningful differences between the classes, as early as 2 weeks on-treatment (Fig. 2a). Complete responders and non-responsive patients were more clearly separated than partially responding patients. These results suggest that there is a potentially greater predictive value looking at on-treatment than pre-treatment biomarkers.

**Table 1:** Summary of patient characteristics for the NEO study and I-SPY validation set

Characteristics	NEO cohort (50)	NEO cohort PT-TM pairs (23)	*p* value	I-SPY 1 PT-T2 pairs (36)	*p* value
Median age at diagnosis	50.8	50.1	0.8	47	
Tumour grade			0.52		0.58
1	0 (0%)	0 (0%)		1 (3%)	
2	22 (44%)	12 (52%)		20 (55%)	
3	28 (56%)	11 (48%)		15 (42%)	
Hormone receptor status			0.24		0.66
Positive	23 (46%)	14 (61%)		24 (67%)	
Negative	27 (54%)	9 (39%)		12 (33%)	
HER2 status			0.87		0.64
Positive	10 (20%)	5 (22%)		6 (17%)	
Negative	40 (80%)	18 (78%)		30 (83%)

### Responding and non-responding tumours are more different upon exposure to chemotherapy

In an attempt to quantify the molecular differences between the response groups at each time point, rank product analysis was performed at different standard *p* values (0.05, 0.01, and 0.001). This approach was hampered by different numbers of samples at each time point (with T2 having very few samples); however, the number of genes differentially expressed at all *p* values tended to be greater during rather than before treatment (Fig. 2b). Similar results were also seen using 1%, 5%, and 10% FDR (Fig. 2b). The biggest differences between the response classes were at TM (mid-chemo), which agrees with the LDFA results, which showed the least amount of overlap of the response classes at TM. Gene set enrichment analysis across the response classes at each time point also demonstrated more enriched pathways after 2 weeks of treatment (29), mid-chemo (30), and resection (29), compared to pre-treatment (18) (Additional file 2: Figure S2A). Next, we sought to examine common differentially expressed genes between responders and non-responders across the two datasets. Far more genes were commonly significantly differentially expressed (FDR = 10% between responders and non-responders on-treatment in the NEO and I-SPY 1 datasets compared with pre-treatment. In accordance with the LFDA results, more significantly differentially expressed genes (1814) were observed between on-treatment samples, with 6% (197), but only one was common between NEO and I-SPY pre-treatment (Additional file 2: Figure S2B and Additional file 4). Examination of the 468 most significantly differentially expressed genes (*p* < 0.001) between responders and non-responders in the NEO dataset at mid-chemo did not clearly distinguish between response groups or time points illustrated by the heatmap in Additional file 3: Figure S3, further demonstrating that identifying biomarkers of response to chemotherapy is very difficult.

We were also keen to evaluate whether the intrinsic subtype assigned to tumours would alter upon treatment. Looking at the NEO and I-SPY datasets, together we found that basal tumours were relatively stable with only 2/19 (11%) tumours changing. More tumours were classified as Luminal A or normal-like on-treatment, which likely reflects a reduction in the expression of proliferation genes during chemotherapy (Fig. 2c).

### AAGAB is a promising potential novel on-treatment biomarker of response to chemotherapy

**Fig. 3:**
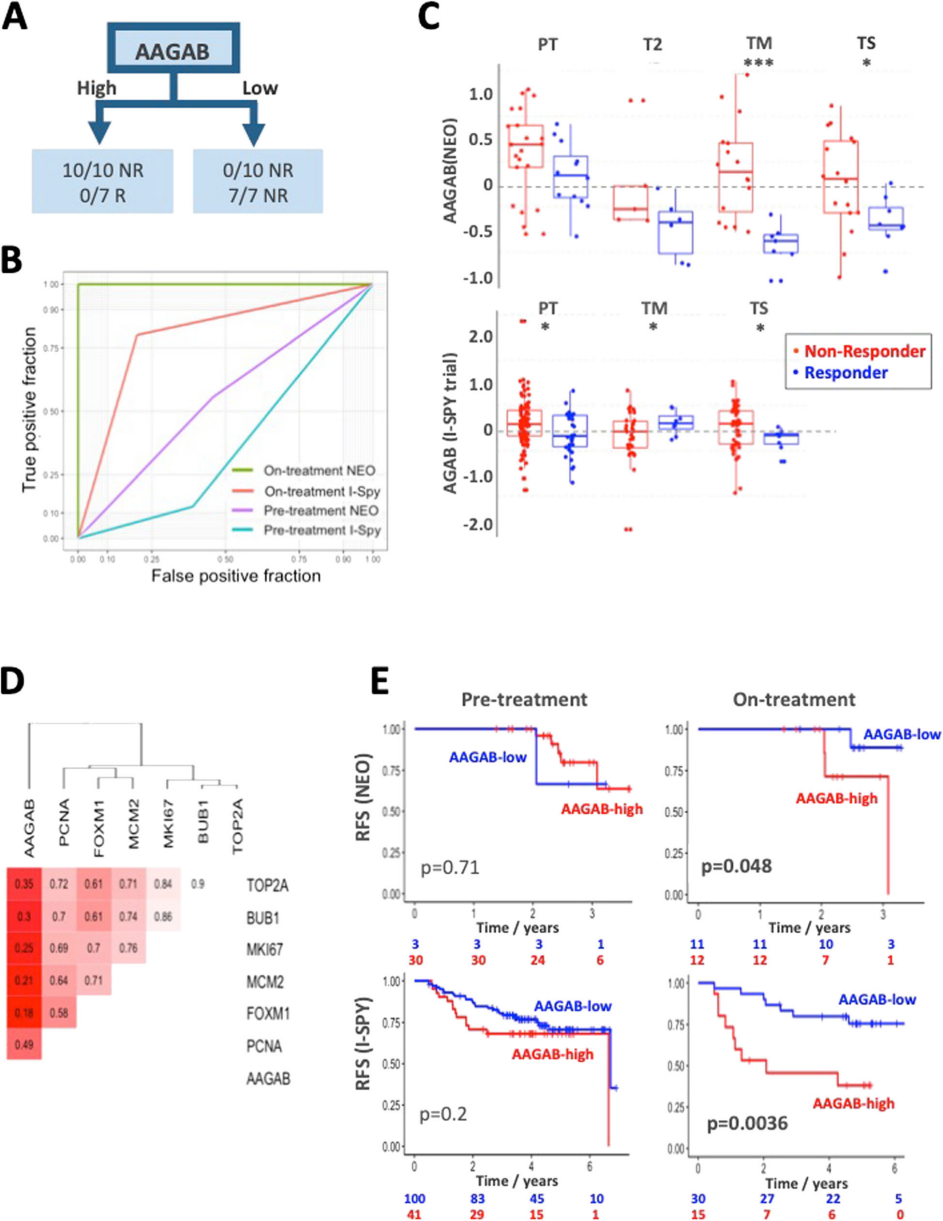
AAGAB is a promising on-treatment biomarker of chemotherapy response and outcome. **a** CART analysis identified AAGAB as a possible biomarker from the Edinburgh NEO dataset and was 100% accurate at predicting pCR in the training data and 76% accurate in the I-SPY 1 validation set. **b** The ROC curves highlight the difference in on-treatment and pre-treatment accuracy and selectivity. **c** Strip charts showing the level of AAGAB in responding and non-responding patients across time points. **d** AAGAB showed no significant (Pearson) correlation with established markers of proliferation in the NEO dataset, indicating it does not seem to be a downstream proxy of their regulation. **e** Kaplan-Meier plots demonstrate that on-treatment, but not pre-treatment, levels of AAGAB were significantly associated with the outcome in both cohorts. *p* values are log-rank test

The mid-chemo gene list from the NEO dataset (1102 genes, unadjusted *p* value = 0.01) was fed to a random forest model for further feature selection and classification and regression tree (CART) model, which reported *AAGAB* as the most predictive gene for response prediction in the NEO training dataset with 100% accuracy for pCR prediction on the mid-chemo samples (Fig. 3a). Validation was conducted completely independently on publicly available sequentially sampled chemotherapy data from the I-SPY 1 Trial [10] and reported 76% accuracy using *AAGAB* at the same expression level on the scaled and centred expression data at the on-treatment time point prior to resection (T2). For comparison, the pre-treatment only sample gene lists were put through the same protocol in order to consider whether highly predictive models could be generated before chemotherapy. IGF1R was the most predictive pre-treatment marker with an accuracy of 74% and 63% in the NEO and I-SPY datasets, respectively (Table 2). AAGAB was the sixth most accurate predictor (65%, 57%); receiver operator curves show the relative specificity and sensitivity of this marker pre- and on-treatment (Fig. 3b). Gene expression levels of AAGAB were lower in responders across all time points in the NEO cohort but were most significantly different at mid-chemo. In the I-SPY dataset, *AAGAB* was significantly lower before treatment and at excision (Fig. 3c). We wondered whether *AAGAB* was lower in responders due to a reduction in proliferation, but Pearson correlation analysis with common proliferation-associated genes (*TOP2A*, *BUB1*, *MKI67*, *MCM2*, *FOXM1*, and *PCNA*) demonstrated no significant correlation to any of these genes (Fig. 3d), suggesting that *AAGAB* is independent of proliferation. Survival analysis demonstrated that response status predicted by *AAGAB* level, at mid chemo in the NEO study and at 2 weeks in the I-SPY 1, was significantly associated with the outcome (NEO *p* = 0.048, I-SPY 1 *p* = 0.0036) (Fig. 3e). Interestingly, the level of *AAGAB* before treatment was not associated with the outcome in either cohort (*p* = 0.71 and *p* = 0.2, Fig. 3e). None of the other top 10 pre- or on-treatment markers was significantly associated with the outcome in both datasets (Table 2); only one gene (*ARF5*) was associated with the outcome in the NEO dataset (*p* = 0.004). Taken together, the single gene on-treatment biomarker AAGAB appears to outperform novel pre-treatment markers and established prognostic tests in predicting pCR and long-term outcome to chemotherapy.

**Table 2:** Comparison of pre- and on-treatment biomarkers for predicting response and outcome. Evaluation of the performance of the top 10 pre- and on-treatment genes identified for predicting pathological response in the NEO dataset

	Response accuracy		Response AUC		Outcome (log-rank)	
	NEO	I-SPY	NEO	I-SPY	NEO	I-SPY
On-treatment						
AAGAB	*100%*	78%	1.00	0.63	**0.048**	**0.0036**
ZNF165	*88%*	54%	0.91	0.57	0.26	0.70
KRTCAP3	*79%*	52%	0.85	0.56	0.81	0.49
RFC2	*79%*	40%	0.85	0.35	0.51	0.44
C20orf151	*70%*	NA	0.75	NA	0.36	NA
ARF5	*70%*	43%	0.75	0.36	**0.0038**	0.20
BSPRY	*70%*	48%	0.75	0.49	0.47	0.19
NGRN	*58%*	NA	0.66	Na	0.53	Na
CHST7	*29%*	46%	0.21	0.52	0.65	0.40
SLC18B1	*25%*	Na	0.18	NA	0.55	NA
Pre-treatment						
IGF1R	*74%*	63%	0.76	0.69	0.36	0.11
CTNNB1	*71%*	49%	0.73	0.46	0.60	0.40
SLC20A2	*71%*	56%	0.72	0.57	0.063	0.56
HMGCL	*68%*	47%	0.67	0.45	0.10	0.97
ST6GALNAC5	*68%*	52%	0.69	0.53	0.6	0.28
AAGAB	*65%*	57%	0.65	0.58	0.71	0.20
C1orf51	*62%*	NA	0.61	NA	0.12	NA
KRTCAP3	*62%*	54%	0.63	0.57	0.78	0.78
SETDB2	*50%*	49%	0.48	0.51	0.29	0.15
FADS2	*29%*	48%	0.27	0.5	0.14	0.73

*NA* not available, gene not represented in I-SPY dataset; *AUC* area under curve. Bold indicates significant *p*-values. Italics indicate training prediction percentages

### Comparison of pre- and on-treatment predictions of response and outcome

**Fig. 4:**
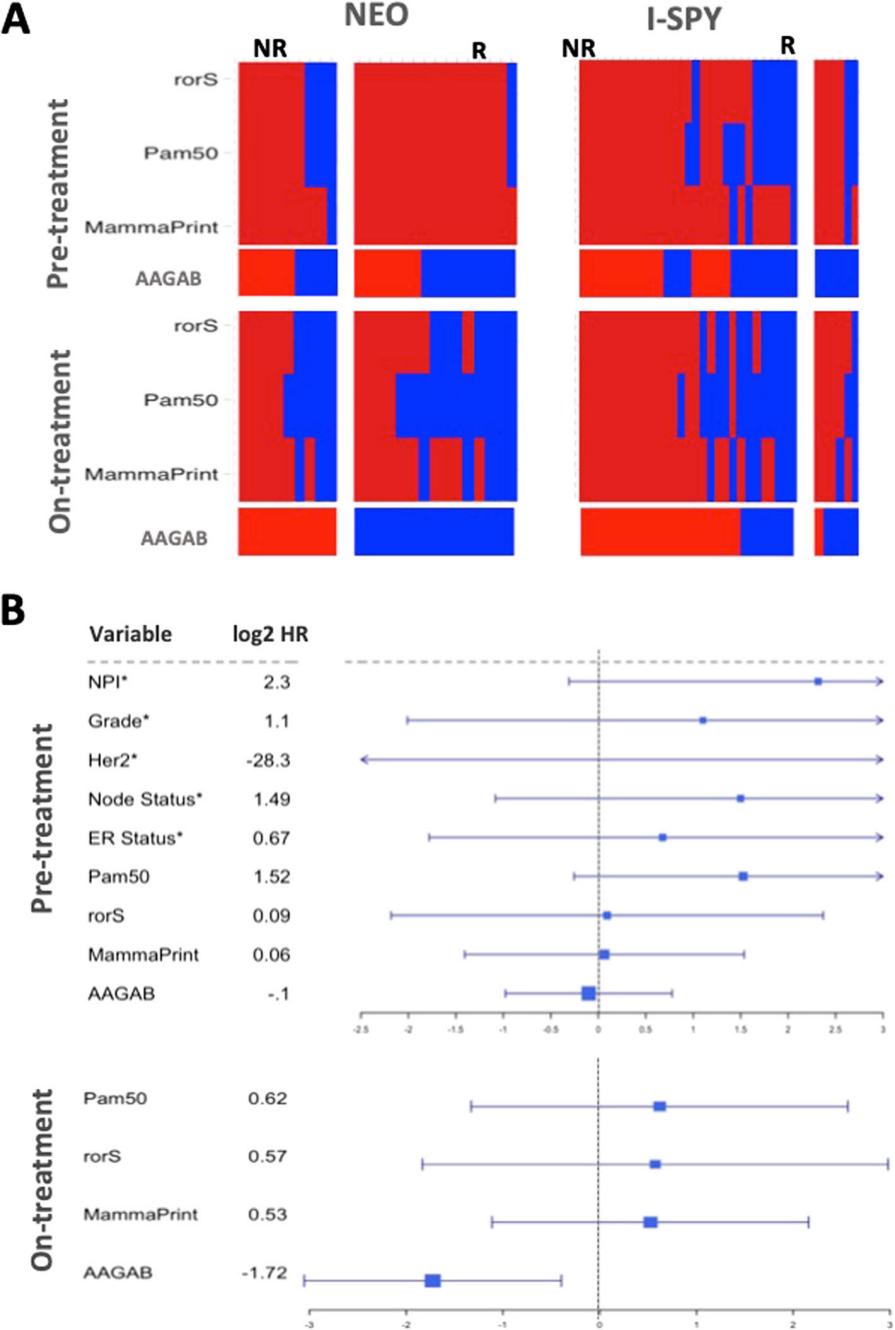
On-treatment signatures more accurately predict pathological response and outcome than pre-treatment. **a** A greater proportion of patients with pathological response are predicted as responders with estimations of molecular signatures on-treatment than pre-treatment. Concordance between patients predicted as high and low risk across time is poor, but the positive predictive value of these tests increase with treatment. For PAM50 subtypes, normal-like and Luminal A are considered good prognosis and basal/Luminal B/HER2-enriched are considered poor outcome. Red = predicted poor outcome, blue = predicted good outcome. **b** Forrest plots to compare molecular signatures and AAGAB before and on-treatment combining both datasets, except where indicated* due to individual sample data unavailable for I-SPY 1 patients

We were also keen to assess whether estimations of established prognostic signatures might be different upon treatment and if on-treatment might be more accurate. All and almost all responding patients were predicted to have poor outcomes with the estimated Mammaprint [19], PAM50 [20], or rorS [21] signatures in pre-treatment samples of the NEO cohort, whereas around half of the responding patients were predicted as good outcome using on-treatment data (Fig. 4a). Overall accuracy improved by 2–8% using on- rather than pre-treatment data; however, improvement in the predictive power of these tests was not uniform between response classes. Good outcome predictions for responders to neoadjuvant chemotherapy saw an aggregate increase in predictive power from 11 to 44.4%, whilst poor outcome predictions for non-responders saw a moderate decrease in accuracy, 75 to 63%. None of the gene expression signatures either pre- or on-treatment or established prognostic markers (NPI, Grade, Her2 status) was significantly associated with the outcome in contrast to the remarkable performance of on-treatment measurement of AAGAB (Fig. 4b).

## Discussion

Determining molecular differences between tumours to select the most effective treatment is the defining feature of precision oncology. Accurately predicting which patients will respond to treatment before exposure relies on a highly specific target. In breast cancer, ER status is a good indicator of response to endocrine treatment, but resistance, both primary and acquired, is common. Chemotherapy is an unselective treatment, relying on cancer cells growing faster than normal cells. The results presented here, along with others [7, 8], suggest on-treatment biomarkers have improved value in predicting whether tumours respond to treatment and are associated with the outcome. Changes in gene expression in sequential patient-matched were fairly consistent in response to chemotherapy across two independent datasets, regardless of the response status. Identifying molecular markers between responding and non-responding tumours was much more challenging. We previously demonstrated that lobular and ductal breast cancers respond to endocrine treatment in the same way, despite clear histological and molecular distinctions that are apparent and maintained on-treatment [22], demonstrating that pre-treatment variations do not necessarily lead to differences in response. The results of this study are somewhat exploratory, rather than definitive, but further illustrate the considerable potential value of on-treatment sampling.

There are no universally agreed-upon markers predictive of response to chemotherapy, and the few that have been investigated in the neoadjuvant setting typically centre around established markers including ER, P53 HER2, and Ki-67 [23]; thus, the introduction of new novel biomarkers can expand the currently available clinical options for physicians. A study published over a decade ago stated that the differences in gene expression between responders and non-responders to neoadjuvant chemotherapy must be rather subtle [12]. The results presented here confirm this statement; however, our results suggest that on-treatment biomarkers may provide important information for predicting response.

As cancer is inherently a proliferative disease, measuring the change in markers of proliferation on-treatment is logical and genes like ki-67 have been demonstrated previously to be potentially a new clinical tool for disease prognosis and prediction [24, 25]. It is therefore all the more interesting that the potentially novel biomarker identified in this study, AAGAB is not tightly correlated with known markers of proliferation. AAGAB has primarily been studied for its role in punctate palmoplantar keratoderma [26] and the role of adaptin in the clathrin-independent endocytosis of epidermal growth factors. The level of AAGAB was found to be prognostic of response (*p* < 0.001) in renal cancers (favourably) and in thyroid cancers (unfavourably) from the TCGA study, and expression is elevated in breast cancer, relative to the normal breast (*p* < 0.001). However, the exact role of AAGAB in breast cancer is currently unclear and potentially warrants further investigation. Clearly, further validation of the role of AAGAB in breast cancer is warranted and will be performed as new neoadjuvant chemotherapy datasets become available. This study supports the use and identification of genes or markers from on-treatment biopsies as a tool for improving patient response classification. We propose that the use of on-treatment samples offers valuable insight into the dynamic changes correlated with response, and submit our findings as support for continued neoadjuvant sampling, and novel biomarker generation.

## Conclusion

We have identified AAGAB as a novel on-treatment biomarker for accurate prediction of pCR and outcome in patients treated with neoadjuvant chemotherapy. A semi-supervised analysis and evaluation of estimations of established molecular signatures also highlight the potential value of on-treatment biomarkers. Combining on-treatment biomarkers with known clinical prognostic factors could further improve the accuracy of response predictions and deserve further study. On-treatment expression changes in the neoadjuvant setting may offer greater possibilities for the identification and creation of more future novel biomarkers.

## Additional files

Additional file 1: **Figure S1.** A, Pairwise significant analysis of microarrays (FDR = 10%) demonstrating that whilst only a relatively small proportion of genes are significantly up- or downregulated in response to chemotherapy in both datasets, overall changes in patient-matched sequential samples response to treatment are highly consistent. Red = upregulated, blue = downregulated on- relative to pre-treatment. Gene lists are in Additional file 4: Table S1. B, Unsupervised principal component analysis cannot distinguish responding from non-responding breast tumours receiving chemotherapy, before or on-treatment. (JPG 126 kb)

Additional file 2: **Figure S2.** A, Gene set enrichment analysis (GSEA) results showing greater numbers of enriched pathways between responders and non-responders on-treatment compared to pre-treatment in the NEO dataset. B, Venn diagrams indicating that there were many more overlapping significantly differentially expressed genes between responders and non-responders across the two studies on-treatment compared to pre-treatment. Gene lists are for FDR = 10% (see Additional file 4: Table S3). (JPG 72 kb)

Additional file 3: **Figure S3.** Heatmap of the 468 most significantly differentially expressed genes (*p* < 0.001) between responders and non-responders in the NEO dataset at mid-chemo, demonstrating rather poor separation between the response groups and time points. Gene list is in Additional file 4: Table S2. (JPG 91 kb)

Additional file 4: **Table S1.** Gene lists of pairwise analysis of pre- and on-treatment sequential patient-matched samples from NEO and I-SPY datasets using Significance analysis of microarrays with FDR = 10%. **Table S2.** Gene lists distinguishing between responders and non-responders at different time points across the NEO and ISPY datasets using rank product analysis with *p* < 0.05, *p* < 0.01, and *p* < 0.001. Pre-treatment (TP), 2 weeks (T2), mid-chemo (TM), and surgery (TS). **Table S3.** Gene lists distinguishing between responders and non-responders at different time points across the NEO and I-SPY datasets using significance analysis of microarrays with FDR = 10%, FDR = 5%, and FDR = 1%. Pre-treatment (TP), 2 weeks (T2), mid-chemo (TM), and surgery (TS). (XLSX 1184 kb)

## Data Availability

Gene expression data associated from the study has been made publicly available at NCBI GEO under the accession number GSE122630.
