# Supplementary figures and images for: On-treatment biomarkers can improve prediction of response to neoadjuvant chemotherapy in breast cancer
Source: Breast Cancer Res. 2019 Jun 14;21:73. doi: 10.1186/s13058-019-1159-3 (PMC6570893; doi:10.1186/s13058-019-1159-3)

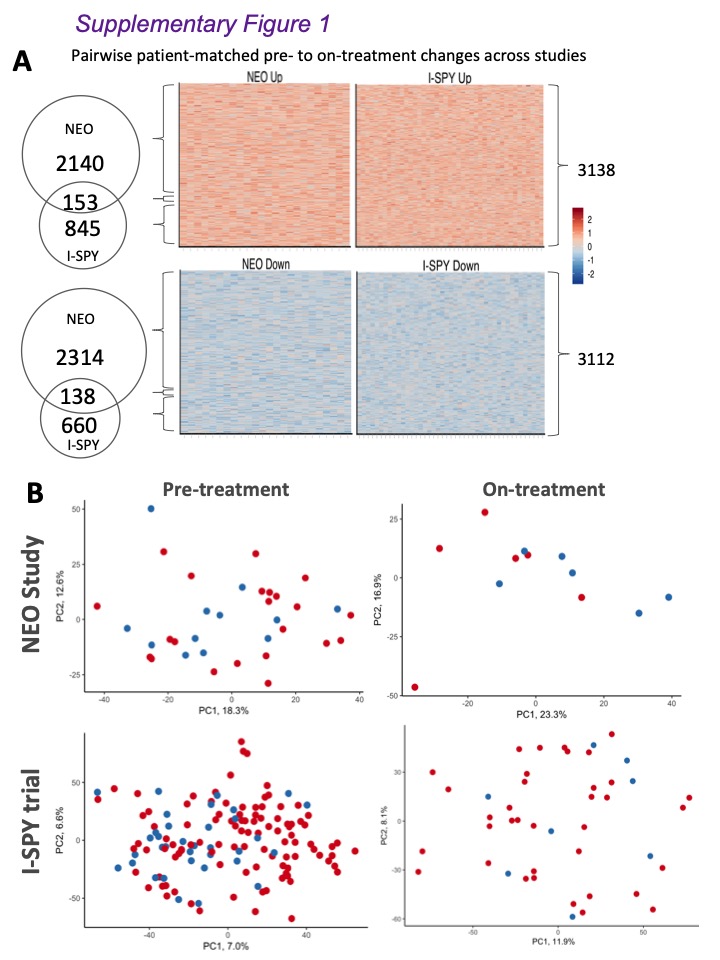

Supplement: Supplementary file 1 — Figure S1. A, Pairwise significant analysis of microarrays (FDR = 10%) demonstrating that whilst only a relatively small proportion of genes are significantly up- or downregulated in response to chemotherapy in both datasets, overall changes in patient-matched sequential samples response to treatment are highly consistent. Red = upregulated, blue = downregulated on- relative to pre-treatment. Gene lists are in Additional file 4: Table S1. B, Unsupervised principal component analysis cannot distinguish responding from non-responding breast tumours receiving chemotherapy, before or on-treatment. (JPG 126 kb) [file 13058_2019_1159_MOESM1_ESM.jpg]

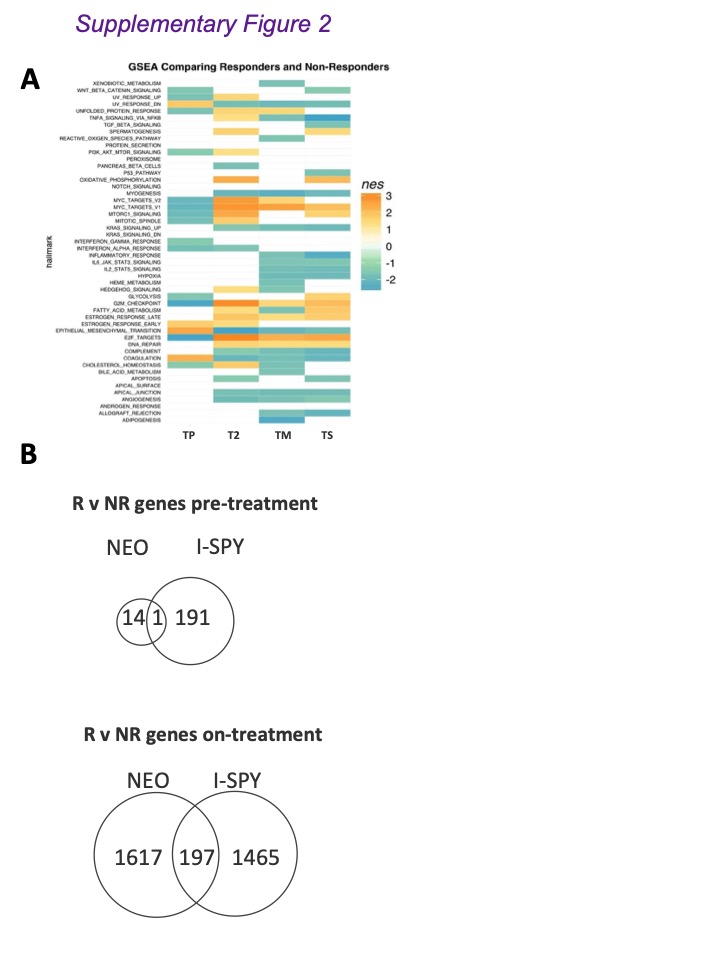

Supplement: Supplementary file 2 — Figure S2. A, Gene set enrichment analysis (GSEA) results showing greater numbers of enriched pathways between responders and non-responders on-treatment compared to pre-treatment in the NEO dataset. B, Venn diagrams indicating that there were many more overlapping significantly differentially expressed genes between responders and non-responders across the two studies on-treatment compared to pre-treatment. Gene lists are for FDR = 10% (see Additional file 4: Table S3). (JPG 72 kb) [file 13058_2019_1159_MOESM2_ESM.jpg]

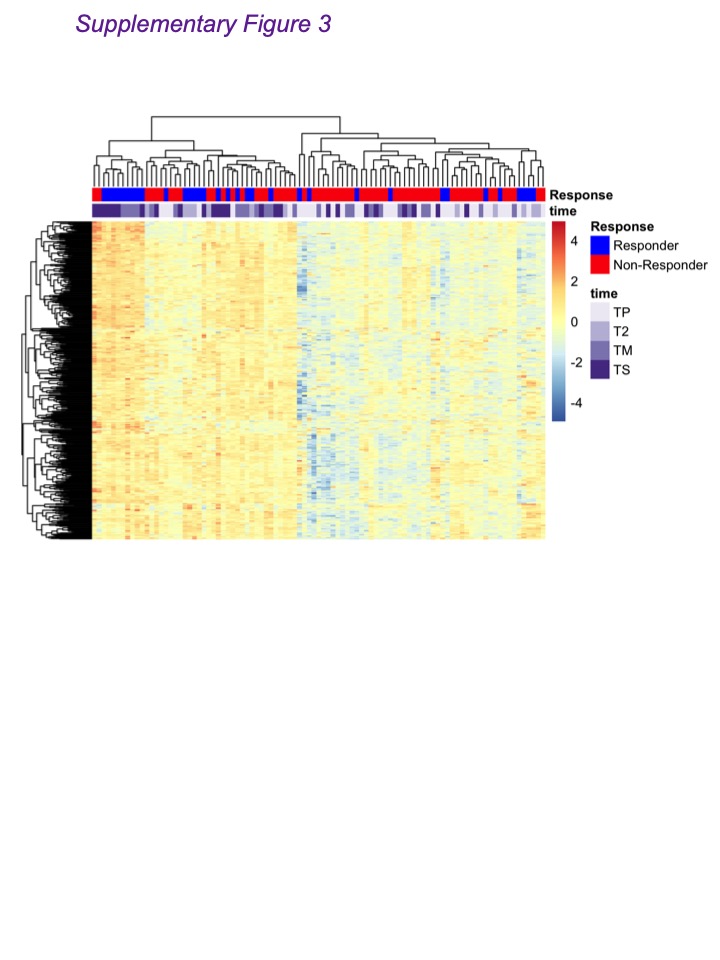

Supplement: Supplementary file 3 — Figure S3. Heatmap of the 468 most significantly differentially expressed genes (p < 0.001) between responders and non-responders in the NEO dataset at mid-chemo, demonstrating rather poor separation between the response groups and time points. Gene list is in Additional file 4: Table S2. (JPG 91 kb) [file 13058_2019_1159_MOESM3_ESM.jpg]
